# STIM Proteins and Orai Ca^2+^ Channels Are Involved in the Intracellular Pathways Activated by TLQP-21 in RAW264.7 Macrophages

**DOI:** 10.3389/fphar.2018.01386

**Published:** 2018-11-27

**Authors:** Laura Molteni, Laura Rizzi, Elena Bresciani, Ramona Meanti, Jean-Alain Fehrentz, Pascal Verdié, Robert J. Omeljaniuk, Giuseppe Biagini, Vittorio Locatelli, Antonio Torsello

**Affiliations:** ^1^School of Medicine and Surgery, University of Milano-Bicocca, Monza, Italy; ^2^CNRS, Max Mousseron Institute of Biomolecules UMR5247, ENSCM, University of Montpellier, Montpellier, France; ^3^Department of Biology, Lakehead University, Thunder Bay, ON, Canada; ^4^Department of Biomedical, Metabolic and Neural Sciences, Laboratory of Experimental Epileptology, University of Modena and Reggio Emilia, Modena, Italy

**Keywords:** TLQP-21, SOCE, STIM-1, macrophages, calcium, receptor, VGF

## Abstract

TLQP-21 is a neuropeptide which has been implicated in regulation of nociception and other relevant physiologic functions. Although recent studies identified C3a and gC1q receptors as targets for TLQP-21, its intracellular molecular mechanisms of action remain largely unidentified. Our aim was (i) to explore the intracellular signaling pathway(s) activated by JMV5656, a novel derivative of TLQP-21, in RAW264.7 macrophages, and (ii) to assess linkages of these pathways with its purported receptors. JMV5656 stimulated, in a dose-dependent fashion, a rapid and transient increase in intracellular Ca^2+^ concentrations in RAW264.7 cells; repeated exposure to the peptide resulted in a lower response, suggesting a possible desensitization mechanism of the receptor. In particular, JMV5656 increased cytoplasmic Ca^2+^ levels by a PLC-dependent release of Ca^2+^ from the endoplasmic reticulum. STIM proteins and Orai Ca^2+^ channels were activated and played a crucial role. In fact, treatment of the cells with U73122 and thapsigargin modulated the increase of intracellular Ca^2+^ levels stimulated by JMV5656. Moreover, in RAW264.7 cells intracellular Ca^2+^ increases did not occur through the binding of JMV5656 to the C3a receptor, since the increase of intracellular Ca^2+^ levels induced by JMV5656 was not affected by specific siRNA against C3aR. In summary, our study provides new indications for the downstream effects of JMV5656 in macrophages, suggesting that it could activate receptors different from the C3aR.

## Introduction

Macrophages, the first line of defense in the host, are found in all tissues and contribute to several physiological functions, including homeostasis, tissue repair, and development (Wynn et al., 2013; Epelman et al., 2014). Macrophages are also known to participate in different pathological phenomena by infiltrating tissues in response to inflammatory stimuli, and releasing pro-inflammatory cytokines (Hashimoto et al., 2013; Ristoiu, 2013). As well, macrophages are involved in the generation and maintenance of neuropathic pain (Clark et al., 2013; Ristoiu, 2013; Willemen et al., 2014), a pathological chronic condition caused by nerve injury, and characterized by increased sensitivity to mechanical and thermal stimuli (Skaper et al., 2012; Pannell et al., 2016). Recently, gene expression profile studies have shown that *vgf* (non-acronymic) is a frequently upregulated gene in several models of neuropathic pain (Moss et al., 2008; Maratou et al., 2009; Riedl et al., 2009; Chen et al., 2013; Lind et al., 2016).

The *vgf* gene was originally identified in PC12 rat pheochromocytoma cells (Levi et al., 1985); its expression is restricted to subpopulations of neurons and neuroendocrine cells (van den Pol et al., 1989). The *vgf* gene encodes a neuropeptide precursor (615 amino acids in human and 617 amino acids in rodents) that is processed by prohormone convertases (PCs) 1/3 and 2 to produce numerous smaller and bioactive peptides. TLQP-21 (VGF^556-576^) is one of the best-studied and characterized VGF-derived fragments, and regulates different biological processes, like energy balance, lipolysis, and gastric functions, as well as reproduction and inflammatory pain (Bartolomucci et al., 2006; Sibilia et al., 2010; Aguilar et al., 2013; Fairbanks et al., 2014). In particular, TLQP-21 was found to activate macrophages through the complement component C1q receptor (gC1qR), causing mechanical hypersensitivity in rats (Chen et al., 2013). Other authors have also reported that TLQP-21, by binding to the complement component C3a receptor (C3aR), has a role in directing migration of macrophages (Hannedouche et al., 2013). Since gC1qR and C3aR are both receptors of the complement system, an integral part of the innate immunity that mediates responses to inflammatory triggers (Nesargikar et al., 2012), the involvement of TLQP-21 in the inflammatory process is suggested.

Recently, a novel derivative of TLQP-21, JMV5656, has been found to retain biological activity, effectively stimulate increases in intracellular calcium (Ca^2+^) levels, and to activate an outward potassium (K^+^) current in microglial cells (Rivolta et al., 2017).

Consequently, the purpose of this study was to investigate intracellular signaling pathways activated by JMV5656 in RAW264.7 cells, and to evaluate whether this peptide could bind to C3aR. The data presented here clearly indicate that in macrophages JMV5656 stimulates intracellular Ca^2+^ release from the endoplasmic reticulum (ER) in a phospholipase C (PLC)-dependent way, most likely by binding a receptor different from C3aR.

## Materials and Methods

### Chemicals

TLQP-21 (TLQPPASSRRRHFHHALPPAR) and JMV5656 (RRRHFHHALPPAR) were synthesized using conventional solid phase synthesis and then purified on a C18 reversed phase column. Each peptide was purified to a purity of at least 96% by high-performance liquid chromatography (HPLC). Prior to assay, peptides were first dissolved in ultrapure water, and then diluted in Hank’s Balanced Salt Solution (HBSS) to final working concentrations. Cyclosporine A (CsA), thapsigargin (TG), U73122, 2-aminoethyl diphenylborinate (2-APB), SKF-96365, YM-58483, and EGTA were purchased from Sigma-Aldrich (St Louis, MO, United States). C3a was purchased from Merck Millipore (Billerica, MA, United States). C3a_(70-77)_ was purchased from D.B.A. Italia (Segrate, Italy).

### Cell Cultures

RAW264.7 murine macrophage cells were cultured in Dulbecco’s Modified Eagle Medium (DMEM; Sigma-Aldrich) supplemented with 10% heat-inactivated fetal bovine serum (FBS), 100 IU/ml penicillin, 100 μg/ml streptomycin, and 2 mM L-glutamine (Euroclone, Pero, Italy) under standard cell culture conditions (37°C, 5% CO_2_).

### Intracellular Calcium Mobilization Assay

RAW264.7 cells were plated at 40,000 cells/well into black walled, clear bottom 96-well plates (Greiner Bio One, Kremsmünster, Austria) and cultured 2 days up to 90–100% confluence. Before assay, the medium was removed and cells were incubated with FLUO-4 NW for 40 min at 37°C and 5% CO_2_ as previously described (Molteni et al., 2017). Fluorescence was monitored every 0.5 s for the preceding 20 s and the 60 s following stimulation using the multilabel spectrophotometer VICTOR^3^ (Perkin Elmer, Waltham, MA, United States) (excitation, 485 nm; emission, 535 nm). Changes in fluorescence corresponded to changes in intracellular calcium levels. TLQP-21, JMV5656, C3a, and C3a_(70-77)_ were diluted in HBSS and injected into the wells by an automated injector system. Where indicated, antagonists and inhibitors were added at different times before the end of the incubation with FLUO-4 NW as previously described (Molteni et al., 2017). Briefly: 2 μM TG, 20 min; 2 μM CsA, 15 min; 10 μM U73122, 10 min; 75 μM 2-APB, 15 min; 10 μM SKF-96365, 20 min; 10 μM YM-58483, 20 min; and 1 mM EGTA, 30 min. Before the end of each experiments, cells were stimulated with 10 μM ATP to control for their viability. All experiments were performed at 37°C and fluorescence values (*F*) were normalized against the baseline acquired immediately before stimulation (*F*_0_).

### siRNA Experiments

C3aR siRNA duplex (sense: 5′-GUGUACCAGUAUUUGUAUAdTdT-3′; antisense: 5′-UAUACAAAUACUGGUACACdTdT-3′) was purchased from Eurofins Genomics (Vimodrone, Italy). To control for non-specific effects of transfection, the negative control group was transfected with K_Ca_3.1 siRNA (sense: 5′-CGGAGAAACACGUGCACAAdTdT-3′; antisense: 5′-UUGUGCACGUGUUUCUCCGdTdT-3′) (Eurofins Genomics). Transfection was performed in a 12-well or 96-well plate (Euroclone), depending on the type of the experiments, using DharmaFECT 4 Transfection Reagent (Thermo-Scientific, Waltham, MA, United States) according to the manufacturer’s protocol. Subsequent experiments were performed 24 h after transfection.

### Reverse Transcriptase-Polymerase Chain Reaction (RT-PCR)

Total RNA was extracted from RAW264.7 cells using EuroGOLD Trifast reagent (Euroclone) and quantified spectrophotometrically using a Nanodrop^®^ND1000 (Thermo-Scientific). Total RNA was transcribed to cDNA using M-MLV Reverse Transcriptase (Invitrogen, Carlsbad, CA, United States). cDNA was amplified by PCR using GoTaq^®^G2 DNA Polymerase (Promega, Madison, WI, United States) in the presence of the following primers (Sigma-Aldrich): C3aR forward: 5′-CCTTCTCCTTGGCTCACCT-3′; C3aR reverse: 5′-AAATACGGGCACACACATCA-3′; GAPDH forward: 5′-GCCATCAACGACCCCTTCATTG-3′; GAPDH reverse: 5′-TCTGTCATGAGGTTGGCTTTCAG-3′. The amplified samples were loaded in equal volumes onto 1% agarose gel in Tris-Acetate-EDTA (TAE) buffer, and then quantified using a Kodak Digital Science^TM^ Image Station 440CF system. C3aR mRNA levels were normalized to GAPDH levels.

### Western Blotting

RAW264.7 cells were rinsed twice with cold phosphate-buffered saline (PBS) and harvested in cold lysis buffer (20 mM Tris-HCl, pH 7.4, 2 mM EDTA, 0.5 mM EGTA) containing a protease inhibitor cocktail (P8340, Sigma-Aldrich). Cell lysates were sonicated on ice and centrifuged at 115,000 ×*g* for 1 h at 4°C. After 15–20 min of incubation on ice, pellets were resuspended in assay buffer (50 mM Tris-HCl, pH 7.4, 2.5 mM EGTA), and quantified by BCA assay (Thermo-Scientific). Equivalent amounts of samples were run on precast 4–12% gradient gels (Twin Helix, Rho, Italy), and transferred to a polyvinylidene difluoride (PVDF) membrane (GE Healthcare, Little Chalfont, United Kingdom). Non-specific sites were blocked with 5% dried fat-free milk dissolved in PBS supplemented with 0.1% Tween-20 (PBS-T) overnight at 4°C. After washes in PBS-T, membranes were incubated with primary antibodies (rabbit anti-C3a receptor polyclonal antibody, 1:700, Bioss Antibodies, Woburn, MA, United States; rabbit anti-actin antibody, 1:1500, Sigma-Aldrich) for 2 h at room temperature (RT). Membranes were then washed with PBS-T and incubated with a peroxidase-coupled secondary antibody (goat anti-rabbit IgG, 1:5000, Thermo-Scientific) for 1 h at RT. Signals were developed with the enhanced chemioluminescence (ECL) system (GE Healthcare) and detected with a Kodak imaging system. Image J software (National Institutes of Health, Bethesda, MD, United States) was used to quantify protein bands. C3aR protein levels were normalized for β-actin levels.

### Statistical Analysis

Values are expressed as mean ± standard error of the mean (SEM). Normality of data distribution was assessed by the Jarque-Bera test. Experiments were independently replicated at least three times. The statistical significance of differences between groups was evaluated with Student’s *t*-test or, when appropriated, by one-way analysis of variance (ANOVA) followed by Tukey’s test. A *p*-value of less than 0.05 was considered significant.

## Results

### JMV5656 Stimulates Intracellular Ca^2+^ Mobilization in RAW264.7 Cells

**FIGURE 1:**
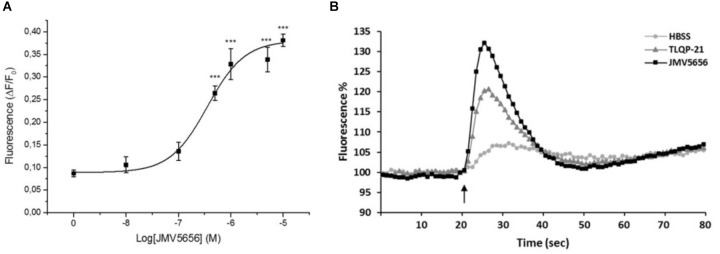
Effect of JMV5656 and TLQP-21 on intracellular Ca^2+^ levels in RAW264.7 cells. Cells were loaded with FLUO-4 NW and fluorescence emissions were measured at 485/535 nm every 0.5 s for the 20 s preceding and the 60 s following the injection of the stimuli. **(A)** JMV5656 was applied at concentrations ranging from 1 nM to 10 μM. Ca^2+^ mobilizing activity is plotted in terms of maximal response obtained at each given concentration in RAW264.7 cells. The 0 was set as the ΔF/F_0_ measured in cells stimulated with the vehicle only. Results are the mean ± SEM of three independent experiments (*n* = 18). ^∗∗∗^*p* < 0.001 vs. 0 concentration (Tukey’s test). **(B)** TLQP-21 and JMV5656 (10 μM) were injected at the time indicated by the arrow. Results are the means of measurements obtained in at least six different wells for each experiment. All experiments were repeated at least three times. One representative experiment is shown.

JMV5656, a novel derivative of TLQP-21, was recently found to be active in microglial cells (Rivolta et al., 2017). In this research, we first tested whether JMV5656 was capable of stimulating an increase of intracellular Ca^2+^ levels in RAW264.7 macrophages. To this aim, cells were stimulated with varied concentrations of JMV5656, ranging from 1 nM to 10 μM. Our results demonstrate that JMV5656 in the concentration range of 500 nM to 10 μM (EC_50_: 0.34 μM) caused a significant rise of intracellular Ca^2+^ levels, reaching a plateau at higher concentrations (Figure 1A). Interestingly, JMV5656 was even more effective than TLQP-21 in inducing an increase in intracellular calcium levels (Figure 1B), supporting the recent findings that the hot spots for TLQP-21 activity reside in its C-terminal region (Cero et al., 2014).

### JMV5656 Stimulates the Release of Ca^2+^ From Intracellular Stores in a PLC-Dependent Way

**FIGURE 2:**
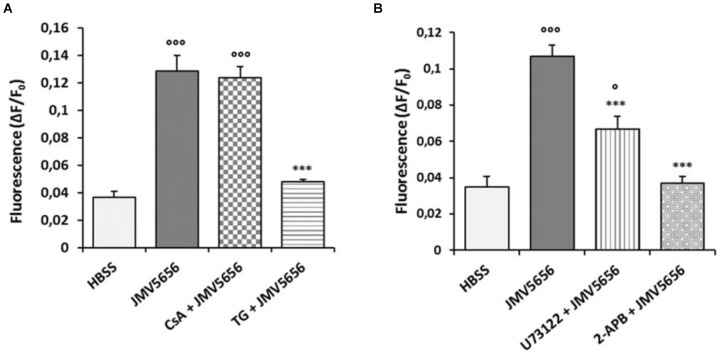
Effects of different inhibitors on JMV5656-mediated intracellular Ca^2+^ increase in RAW264.7 cells. Cells were loaded with FLUO-4 NW and treated with different inhibitors before the stimulation with 1 μM JMV5656. Graphs show intracellular Ca^2+^ mobilization (expressed as fluorescence intensity) in RAW264.7 cells stimulated with **(A)** HBSS (vehicle), JMV5656 alone and JMV5656 in presence of 2 μM CsA (15 min) or 2 μM TG (20 min); and **(B)** HBSS (vehicle), JMV5656 alone, and JMV5656 in presence of 10 μM U73122 (10 min) or 75 μM 2-APB (15 min). Data are shown as the mean ± SEM of measurements obtained in three independent experiments (*n* = 18). °*p* < 0.05, ^∘∘∘^*p* < 0.001 vs. HBSS; ^∗∗∗^*p* < 0.001 vs. JMV5656 (Tukey’s test).

A universal and well-established mechanism for Ca^2+^ signaling is its release from intracellular compartments, where Ca^2+^ ions are held in reserve (Clapham, 2007). We previously demonstrated that in CHO cells TLQP-21 induced an increase of cytoplasmic Ca^2+^ levels through the release of Ca^2+^ from the ER (Molteni et al., 2017). Starting from this observation, we investigated the intracellular transduction pathways activated by JMV5656 in RAW264.7 cells. The involvement of mitochondria was ascertained using CsA, an inhibitor of the mitochondrial permeability transition pore (mPTP). The incubation of RAW264.7 cells with 2 μM CsA did not modify either basal fluorescence (Supplementary Figure S1) nor the increase of intracellular Ca^2+^ levels induced by JMV5656 (Figure 2A). These results suggest that the increase in intracellular calcium does not depend on release from mitochondrial stores. To evaluate the possibility that Ca^2+^ released from the ER could also be involved in the mechanism of action of JMV5656, we measured the effects of the peptide in the presence of TG, an inhibitor of the sarco/endoplasmic reticulum Ca^2+^ ATPase (SERCA). The treatment with 2 μM TG did not modify basal levels (Supplementary Figure S1) but caused a significant reduction of intracellular Ca^2+^ mobilization induced by JMV5656 (about 63% reduction; Figure 2A), confirming that JMV5656 stimulates the release of Ca^2+^ from the ER, but not from mitochondria. Our results also indicate that Ca^2+^ mobilization stimulated by JMV5656 is triggered by the activation of phospholipase C (PLC), since the treatment of RAW264.7 cells with the antagonist U73122 (10 μM) induced a partial, approximately 38%, but significant decrease in JMV5656-induced Ca^2+^ release from the ER (Figure 2B). As a further validation, RAW264.7 cells were incubated with 2-APB, that inhibits inositol-1,4,5-trisphosphate receptor (IP_3_R)-mediated Ca^2+^ release from the ER. As shown in Figure 2B, treatment with 75 μM 2-APB caused a reduction of intracellular Ca^2+^ mobilization induced by JMV5656 of about 65% in RAW264.7 cells. Again, neither U73122 nor 2-APB modified basal fluorescence (Supplementary Figure S1).

### JMV5656 Effects on the Store-Operated Calcium Entry Process (SOCE)

**FIGURE 3:**
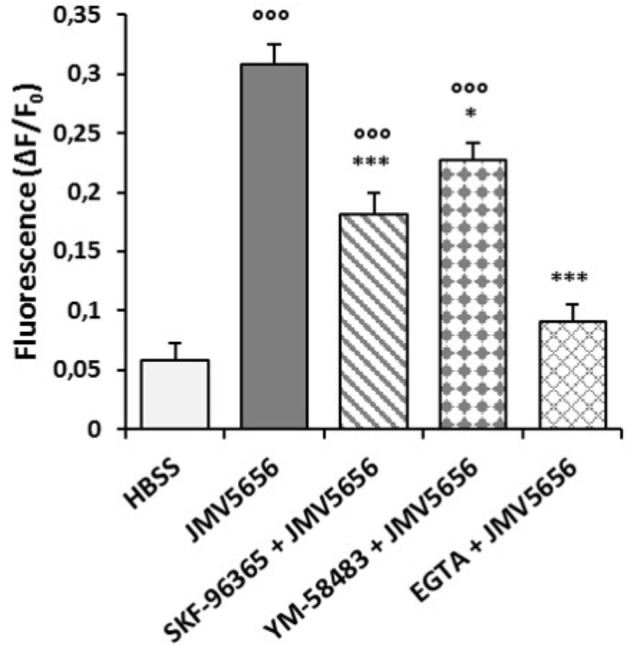
Modulation of intracellular Ca^2+^ levels by JMV5656 in the presence of SOCE antagonists. Cells were loaded with FLUO-4 NW and treated with different inhibitors before stimulation with 1 μM JMV5656. The inhibitors of the SOCE process, SKF96365 (10 μM–20 min), YM-58483 (10 μM–20 min) and EGTA (1 mM–30 min), significantly affected JMV5656-mediated Ca^2+^ mobilization in RAW264.7 cells. Results are shown as the means ± SEM of measurements obtained in three independent experiments (*n* = 18). ^∘∘∘^*p* < 0.001 vs. HBSS; ^∗^*p* < 0.05, ^∗∗∗^*p* < 0.001 vs. JMV5656 (Tukey’s test).

It is known that Ca^2+^ depletion from the ER activates Ca^2+^ entry from outside of the cell through the interaction of stromal interaction molecule (STIM) proteins and Orai proteins (Hewavitharana et al., 2007). To evaluate the involvement of this pathway in JMV5656 activity, we pre-incubated RAW264.7 cells with 10 μM SKF-96365 and 10 μM YM-58483, two specific STIM- and Orai-blockers. Both compounds did not alter basal fluorescence (Supplementary Figure S2) but caused a drop in JMV5656-mediated Ca^2+^ response of about 41% and 26%, respectively (Figure 3). Moreover, the treatment of RAW264.7 cells with the extracellular Ca^2+^ chelator EGTA (1 mM) inhibited the rise in intracellular Ca^2+^ concentration by 71% (Figure 3), suggesting that Ca^2+^ entry from the extracellular environment is an important step in the transduction mechanisms activated by JMV5656. At the end of each experiment, cell viability was tested by stimulation with 10 μM ATP (Supplementary Figures S3–S5).

### JMV5656 Induces a Homologous Desensitization Mechanism in RAW264.7 Cells

**FIGURE 4:**
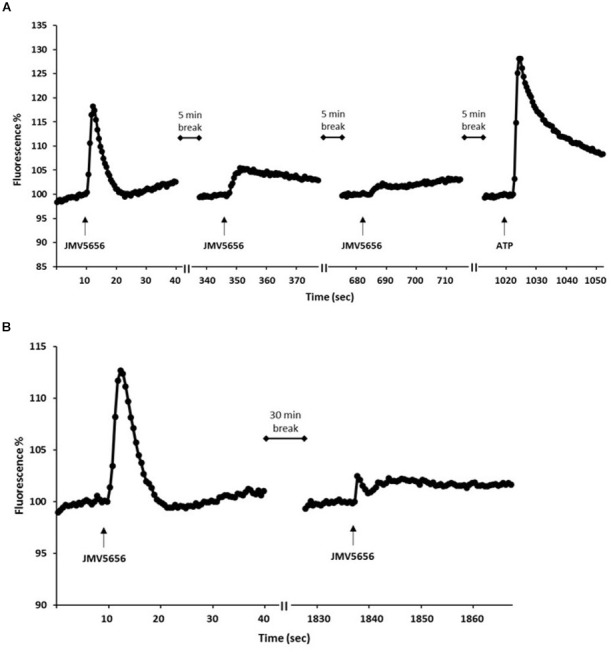
Effects of multiple stimulations with JMV5656 on intracellular Ca^2+^ levels in RAW264.7 cells. Cells were loaded with FLUO-4 NW and fluorescence emissions were measured every 0.5 s for the 30 s following injection of the stimuli. **(A)** The injection of 1 μM JMV5656 was applied three times at 5 min intervals. At the end of experiments, a stimulation with 10 μM ATP was used to check for cell viability. **(B)** 1 μM JMV5656 was applied twice at 30 min interval. Results are the means of measurements obtained in at least six different wells for each experiment. All experiments were repeated at least three times. One representative experiment is shown.

In a second series of experiments, we investigated whether RAW264.7 cells could respond to repeated JMV5656 stimulations given at 5 min intervals. The first administration of 1 μM JMV5656, as expected, induced a robust increase of intracellular Ca^2+^ concentration (Figure 4A). By comparison, second application of 1 μM JMV5656, 5 min later, stimulated only a blunted increase in intracellular Ca^2+^ levels. JMV5656 applied a third consecutive time had no effect on cytoplasmic Ca^2+^ levels (Figure 4A). Subsequent inoculation of cells with 10 μM ATP, 5 min after the third stimulation with JMV5656, induced a robust increment in cell fluorescence, indicating that cells were still viable (Figure 4A). In order to investigate the possibility that 5 min was insufficient for cells to recover from the first administration of JMV5656, a separate independent experiment was conducted in which a second stimulation with JMV5656 was applied 30 min after the first one. Again, 1 μM JMV5656 injected 30 min after the first stimulation failed to induce an increase of intracellular Ca^2+^ levels (Figure 4B), indicating a possible desensitization mechanism of the receptor after repeated stimulation with the peptide.

### C3a and C3a_(70-77)_ Effects on Intracellular Ca^2+^ Levels in RAW264.7 Cells

**FIGURE 5:**
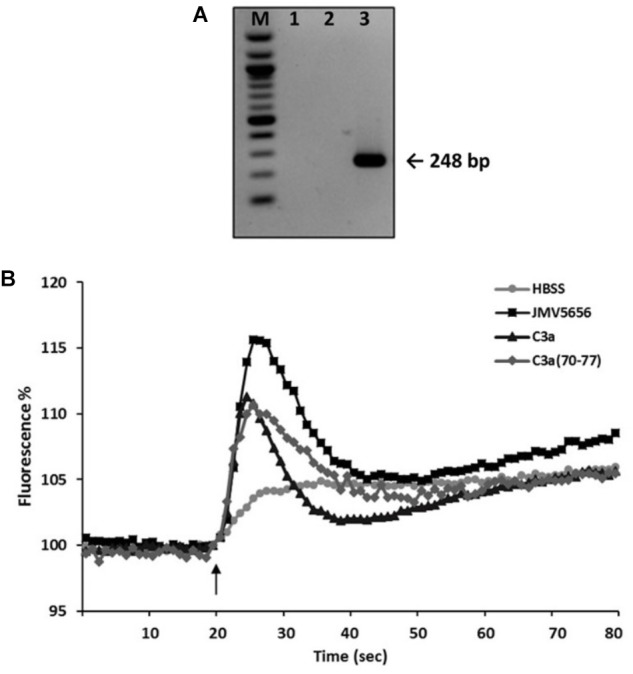
Effect of C3a and C3a_(70-77)_ on intracellular Ca^2+^ levels in RAW264.7 cells. **(A)** Representative PCR amplification for C3aR in RAW264.7 cells. Lane M: molecular weight marker; lane 1: blank, no template control; lane 2: negative control (N-38 cells); lane 3: RAW264.7 cells. **(B)** Cells were loaded with FLUO-4 NW and fluorescence emissions were measured at 485/535 nm every 0.5 s for the 20 s preceding and the 60 s following the injection of the stimuli. HBSS, JMV5656, C3a, and C3a_(70-77)_ (1 μM) were injected at the time indicated by the arrow. Results are the means of measurements obtained in at least six different wells for each experiments. All experiments were repeated at least three times. One representative experiment is shown.

Recently, C3aR has been identified as the target for TLQP-21 in macrophages and ovary cells (Hannedouche et al., 2013). We investigated (i) the presence of the C3aR, and (ii) the activity of C3a and C3a_(70-77)_ peptides in RAW264.7 cells. RT-PCR demonstrated that RAW264.7 cells expressed high levels of C3aR mRNA (Figure 5A), and that both C3a and C3a_(70-77)_ (1 μM) induced an increase of intracellular Ca^2+^ levels (Figure 5B). However, 1 μM JMV5656 was able to stimulate a greater increase of intracellular Ca^2+^ mobilization compared to both C3a peptides (Figure 5B). Since the stimulation of Ca^2+^ levels induced by C3a and C3a_(70-77)_ was almost superimposable, we decided to use C3a_(70-77)_ for the next experiments.

### C3a_(70-77)_ Does Not Induce a Desensitization Mechanism in RAW264.7 Cells

**FIGURE 6:**
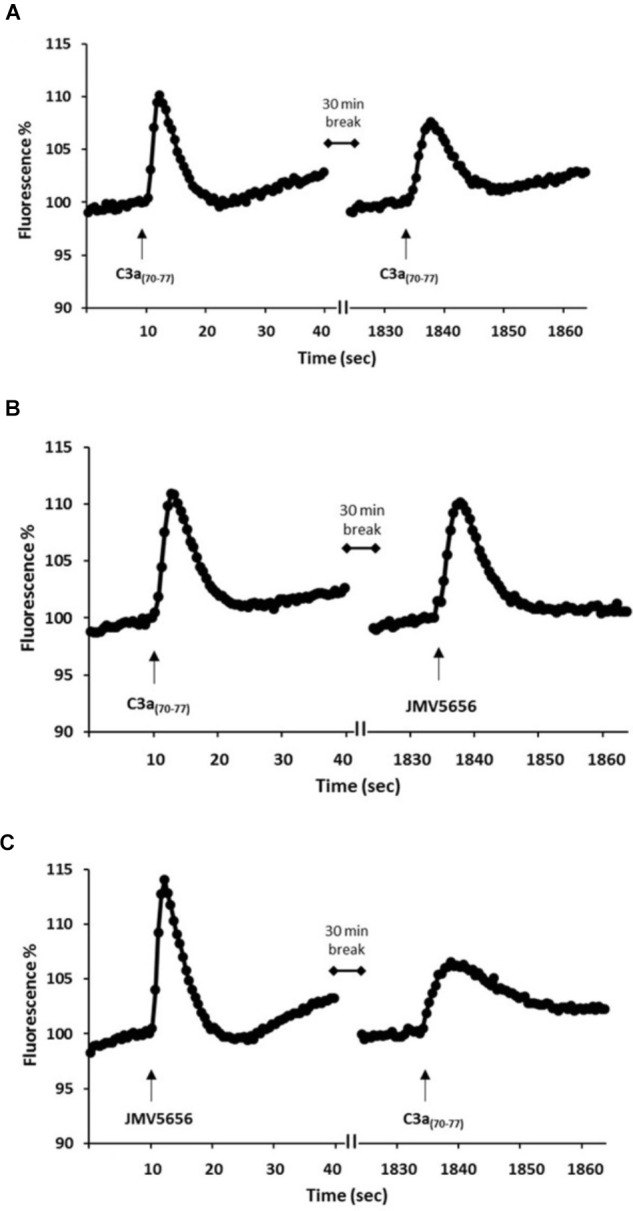
Repeated C3a_(70-77)_ and JMV5656 stimulations on intracellular Ca^2+^ levels in RAW264.7 cells. Cells were loaded with FLUO-4 NW and fluorescence emissions were measured every 0.5 s for the 30 s following the injection of the stimuli. **(A)** 1 μM C3a_(70-77)_ was applied twice at 30 min interval. JMV5656 **(B)** and C3a_(70-77)_ **(C)** were applied after 30 min from C3a_(70-77)_ and JMV5656, respectively. Results are the means of measurements obtained in at least six different wells for each experiments. All experiments were repeated at least three times. One representative experiment is shown.

In order to investigate whether C3a_(70-77)_ induced, like JMV5656, a desensitization mechanism of its receptor, RAW264.7 cells were stimulated with repeated administration of C3a_(70-77)_ given at 30 min interval. Intracellular Ca^2+^ levels rose significantly after the first administration of 1 μM C3a_(70-77)_ (Figure 6A). After 30 min, a second stimulation with the peptide still induced a significant increase of intracellular Ca^2+^ levels (Figure 6A), suggesting that in these cells C3aR does not undergo to a desensitization process upon C3a stimulation. No cross-desensitization was observed when 1 μM JMV5656 was applied 30 min after C3a_(70-77)_ (Figure 6B). In fact, JMV5656 administered as a second stimulation after C3a_(70-77)_ induced a robust increase in cell fluorescence (Figure 6B). Interestingly, the second stimulation with C3a_(70-77)_, applied 30 min after JMV5656, induced a lower but significant increase of intracellular Ca^2+^ levels (Figure 6C) in the cells. These data suggest that in RAW264.7 cells JMV5656 and C3a_(70-77)_ bind to two different receptors.

### JMV5656 Effects Are Not Mediated by C3aR in RAW264.7 Cells

**FIGURE 7:**
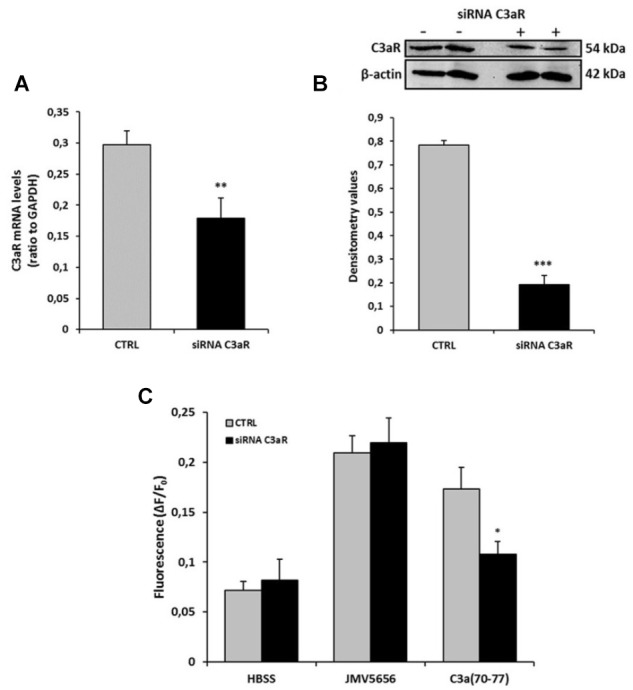
Effect of C3aR siRNA on RAW264.7 cells stimulated with JMV5656 and C3a_(70-77)_. Graphs show mRNA **(A)** and protein **(B)** levels of C3aR after transfection with 25 mM siRNA for 24 h. Data are shown as the mean ± SEM of measurements obtained in at least three independent experiments. ^∗∗^*p* < 0.01, ^∗∗∗^*p* < 0.001 vs. control (CTRL). **(C)** After 24 h from transfection with 25 mM siRNA, cells were loaded with FLUO-4 NW and fluorescence emissions were measured at 485/535 nm every 0.5 s for the 20 s preceding and the 60 s following the injection of the stimuli. Graphs show intracellular Ca^2+^ levels (expressed as fluorescence intensity) in RAW264.7 cells stimulated with HBSS (vehicle), JMV5656, and C3a_(70-77)_ in the absence or presence of C3aR siRNA. Results are shown as the mean ± SEM of measurements obtained in three independent experiments (*n* = 18). ^∗^*p* < 0.05 vs. C3a CTRL (Student’s *t*-test).

To confirm the role of C3aR in the transduction pathway activated by JMV5656, we used specific siRNA to inhibit the expression of C3aR in RAW264.7 cells. Transfection of 25 mM C3aR siRNA duplex for 24 h significantly reduced C3aR mRNA (Figure 7A) and protein levels (Figure 7B). Moreover, siRNA against C3aR reduced the increase of intracellular Ca^2+^ levels mediated by 1 μM C3a of about 38%, while no reduction was observed after 1 μM JMV5656 Ca^2+^ stimulation (Figure 7C), further indicating that JMV5656 induces an increase of intracellular Ca^2+^ levels by binding a receptor different from the C3aR in RAW264.7 cells.

## Discussion

The interest for TLQP-21 as a potential drug is continuously expanding thanks to its involvement in several physiologic functions, like energy homeostasis and nociception (Bartolomucci et al., 2006; Chen et al., 2013; Fairbanks et al., 2014). Different receptors have been identified as targets for TLQP-21 (Chen et al., 2013; Hannedouche et al., 2013), and recently we characterized its intracellular signaling pathway in CHO cells (Molteni et al., 2017). Additionally, we found that JMV5656, a short analog of TLQP-21, activates a Ca^2+^-dependent increase in outward K^+^ current in microglial cells (Rivolta et al., 2017). Consequently, our present study investigates molecular mechanisms of action of JMV5656 in a macrophage cell line. Our results demonstrate that in RAW264.7 cells JMV5656 induces a transient increase in free intracellular Ca^2+^. In immune cells, Ca^2+^ plays an important role for activation of the immune response. In particular, in macrophages Ca^2+^ has been shown to regulate cytokine- and nitric oxide-synthesis, as well as phagocytosis (Vaeth et al., 2015). In our experimental setting, JMV5656, in a dose-dependent manner, significantly stimulated a rapid and transient increase in intracellular Ca^2+^ in RAW264.7 cells, comparably with reports in ovary and immune cells (Cassina et al., 2013; Chen et al., 2013; Molteni et al., 2017; Rivolta et al., 2017).

Our results also indicate that increases of intracellular Ca^2+^ levels induced by JMV5656 are a consequence of the release of Ca^2+^ from the endoplasmic reticulum, as shown by the treatment of RAW264.7 cells with TG, an inhibitor of SERCA. Moreover, in RAW264.7 cells the release of Ca^2+^ from the reticulum is triggered by the activation of PLC, since treatment of the cells with the PLC-antagonist U73122 induced a significant decrease of JMV5656-stimulated intracellular Ca^2+^ release. Indeed, U73122 has been shown to inhibit the hydrolysis of phosphatidylinositol (PPI) to IP_3_ leading to a decrease in free cytosolic Ca^2+^ (Yule and Williams, 1992).

PLCs are known to cleave phosphatidylinositol-4,5-bisphosphate (PIP2) into diacylglycerol (DAG) and IP_3_. DAG activates protein kinase C (PKC), while the binding of IP_3_ to its receptor triggers the release of Ca^2+^ ions from the ER. Our results demonstrate that the treatment of RAW264.7 cells with 2-APB, an IP_3_R antagonist, greatly reduced the increase of intracellular Ca^2+^ levels induced by JMV5656, further confirming that the binding of the peptide with its receptor causes the release of Ca^2+^ from the ER in a PLC-dependent way.

Following depletion of ER Ca^2+^, STIM proteins are activated and translocate to region of the ER in close proximity with the plasma membrane, where they activate Orai Ca^2+^ channels generating a Ca^2+^ release-activated Ca^2+^ (CRAC) current (Soboloff et al., 2012). The SOCE mechanism is common in macrophages, where Ca^2+^ influx is supposed to be linked to the production of reactive oxygen species (ROS) and to be necessary for the engulfment of apoptotic cells (Desai and Leitinger, 2014). In RAW264.7 cells, we observed a reduction of JMV5656 Ca^2+^ stimulation following the treatment of the cells with SKF-96365 and YM-58483, two specific blockers for STIM and Orai, respectively. Moreover, also the treatment with the extracellular Ca^2+^ chelator EGTA reduced the increase of intracellular Ca^2+^ concentration induced by the peptide. These data suggest that extracellular Ca^2+^ represents an important step in the signaling pathway activated by JMV5656 but is not necessary for JMV5656 activity since intracellular Ca^2+^ mobilization is still observed. Extracellular Ca^2+^ influx, however, has been proposed to be essential for macrophages activation, including immunity to infections, phagocytosis, NLRP3 inflammasome activation, and cytokines production (Rossol et al., 2012; Félix et al., 2013; Liu et al., 2016). In this respect, further studies are needed to better understand the role of JMV5656 in mediating the activation of NLRP3 inflammasome pathway and the resulting immune response.

Evidence suggests that SOCE activity could be modulated by the interaction between Orai and Ca^2+^-activated K^+^ (KCa) channels (Chen et al., 2016). Indeed, it has been shown that persistent Ca^2+^ influx into the cell through Orai channels requires an electrical driving force that is sustained by efflux of K^+^ ions (Lin et al., 2014). These data are in agreement with our recent demonstration that JMV5656 is capable of triggering the activation of a Ca^2+^-dependent K^+^ outward current in N9 microglial cells (Rivolta et al., 2017). The opening of K^+^ channels on the plasma membrane is important for its hyperpolarization that permits the persistence of the driving force for Ca^2+^ to entry from the extracellular environment to replenish the intracellular Ca^2+^ stores (Rivolta et al., 2017).

Repeated administration of JMV5656 to RAW264.7 cells caused a decreased calcium response, suggesting a possible desensitization mechanism for JMV5656 receptor. In particular, JMV5656 induced a homologous desensitization to subsequent JMV5656 stimulation given at 5 or 30 min intervals, but not to ATP, indicating that the desensitization only occurred on specific pathways.

Recently, C3aR has been indicated as possible receptor for TLQP-21 in rodents (Hannedouche et al., 2013). C3aR is a G protein-coupled receptor (GPCR) expressed in lung, spleen, ovary, and intestine. Its expression has been reported also on myeloid cells, such as dendritic cells, macrophages and microglia, and on neurons, where it may have a role in central nervous system (CNS) inflammation and during development (Klos et al., 2013). Accordingly, our results revealed the expression of C3aR in RAW264.7 cells. Moreover, C3a and C3a_(70-77)_ peptides induced an increase of intracellular Ca^2+^ levels in these cells. However, unlike JMV5656, repeated administration of C3a_(70-77)_ did not induce a desensitization mechanism, suggesting the existence of two different receptors for these peptides in RAW264.7 cells. Indeed, in our experimental setting, incubation with siRNA against C3aR decreased intracellular Ca^2+^ mobilization induced by C3a but not by JMV5656, confirming the presence of a receptor different from C3aR, and still unidentified, in RAW264.7 cells for JMV5656. Indeed the RAW264.7 also express significant amounts of mRNA for gC1qR, which has been proposed to mediate the effects of TLQP-21 in macrophages (Chen et al., 2013). We could speculate that JMV5656 is capable to stimulate both C3aR and gC1qR and possibly other receptor species, whereas the C3a can stimulate only its specific receptor. Further studies are needed to clarify how many receptors TLQP-21 can bind and which ones are primarily involved in its biologic activities.

## Conclusion

Our research provides new evidence for the downstream effect of the binding of JMV5656 to its receptor in macrophages, and suggests the existence of different receptors for this peptide in rodent cells. Improving knowledge about the intracellular pathway activated by JMV5656 and the identification of the human receptor could help to improve the treatment of several human disorders, including neuropathic pain.

## Ethics Statement

The study was made *in vitro* using cell lines only. No experiments were performed on animals or involved human beings. Approval from the local Ethic Committee or other regulatory agencies was not required.

## Author Contributions

AT, GB, VL, and RO supervised the entire project, designed the research, and wrote the paper. LM, LR, EB, RM, J-AF, and PV conceived and designed the experiments, performed the research, interpreted and analyzed the data. AT, GB, RO, and VL analyzed the data and critically revised the manuscript.

## Conflict of Interest Statement

The authors declare that the research was conducted in the absence of any commercial or financial relationships that could be construed as a potential conflict of interest.

## Supplementary Material

FIGURE S1 Effects of different inhibitors on basal fluorescence in RAW264.7 cells. Cells were loaded with FLUO-4 NW and treated with different inhibitors before the stimulation with HBSS. The lines show intracellular Ca^2+^ mobilization in RAW264.7 cells stimulated with HBSS alone and in presence of 2 μM CsA (15 min), 2 μM TG (20 min), 10 μM U73122 (10 min), or 75 μM 2-APB (15 min). HBSS was injected at the time indicated by the arrow. Results are the means of measurements obtained in at least six different wells for each experiment. All experiments were repeated three times. One representative experiment is shown.

FIGURE S2 Modulation of basal fluorescence in the presence of SOCE antagonists in RAW264.7 cells. Cells were loaded with FLUO-4 NW and treated with different inhibitors before the stimulation with HBSS. The lines show intracellular Ca^2+^ mobilization in RAW264.7 cells stimulated with HBSS alone and in presence of 10 μM SKF-96365 (20 min), 10 μM YM-58483 (20 min), or 1 mM EGTA (30 min). HBSS was injected at the time indicated by the arrow. Results are the means of measurements obtained in at least six different wells for each experiments. All experiments were repeated three times. One representative experiment is shown.

FIGURE S3 Effects of repeated stimulations on intracellular Ca^2+^ levels in RAW264.7 cells. Cells were loaded with FLUO-4 NW and fluorescence emissions were measured at 485/535 nm every 0.5 s for the 20 s preceding and the 60 s following the injection of the stimuli. 10 μM ATP was applied after 30 min from HBSS. HBSS and ATP were injected at the time indicated by the arrow. Results are the means of measurements obtained in at least six different wells for each experiments. All experiments were repeated three times. One representative experiment is shown.

FIGURE S4 Repeated JMV5656 and ATP stimulations on intracellular Ca^2+^ levels in RAW264.7 cells. Cells were loaded with FLUO-4 NW and treated with different inhibitors (2 μM TG 20 min, 10 μM U73122 10 min, 75 μM 2-APB 15 min) before the stimulation with 1 μM JMV5656. 10 μM ATP was applied after 30 min from JMV5656. JMV5656 and ATP were injected at the time indicated by the arrow. Results are the means of measurements obtained in at least six different wells for each experiment. All experiments were repeated three times. One representative experiment is shown.

FIGURE S5 Repeated JMV5656 and ATP stimulations on intracellular Ca^2+^ levels in the presence of SOCE antagonists in RAW264.7 cells. Cells were loaded with FLUO-4 NW and treated with different inhibitors (10 μM SKF-96365 20 min, 10 μM YM-58483 20 min, 1 mM EGTA 30 min) before the stimulation with 1 μM JMV5656. 10 μM ATP was applied after 30 min from JMV5656. JMV5656 and ATP were injected at the time indicated by the arrow. Results are the means of measurements obtained in at least six different wells for each experiment. All experiments were repeated three times. One representative experiment is shown.

## References

[B1] Aguilar E. Pineda R. Gaytán F. Sánchez-Garrido M. A. Romero M. Romero-Ruiz A. (2013). Characterization of the reproductive effects of the Vgf-derived peptide TLQP-21 in female rats: in vivo and in vitro studies. *Neuroendocrinology* 98 38–50. 10.1159/000350323 23485923

[B2] Bartolomucci A. La Corte G. Possenti R. Locatelli V. Rigamonti A. E. Torsello A. (2006). TLQP-21, a VGF-derived peptide, increases energy expenditure and prevents the early phase of diet-induced obesity. *Proc. Natl. Acad. Sci. U.S.A.* 103 14584–14589. 10.1073/pnas.0606102103 16983076 PMC1600003

[B3] Cassina V. Torsello A. Tempestini A. Salerno D. Brogioli D. Tamiazzo L. (2013). Biophysical characterization of a binding site for TLQP-21, a naturally occurring peptide which induces resistance to obesity. *Biochim. Biophys. Acta* 1828 455–460. 10.1016/j.bbamem.2012.10.023 23122777

[B4] Cero C. Vostrikov V. V. Verardi R. Severini C. Gopinath T. Braun P. D. (2014). The TLQP-21 peptide activates the G-protein-coupled receptor C3aR1 via a folding-upon-binding mechanism. *Structure* 22 1744–1753. 10.1016/j.str.2014.10.001 25456411 PMC4353613

[B5] Chen M. Li J. Jiang F. Fu J. Xia X. Du J. (2016). Orai1 forms a signal complex with BKCa channel in mesenteric artery smooth muscle cells. *Physiol. Rep.* 4:e12682. 10.14814/phy2.12682 26755740 PMC4760400

[B6] Chen Y. C. Pristera A. Ayub M. Swanwick R. S. Karu K. Hamada Y. (2013). Identification of a receptor for neuropeptide VGF and its role in neuropathic pain. *J. Biol. Chem.* 288 34638–34646. 10.1074/jbc.M113.510917 24106277 PMC3843076

[B7] Clapham D. E. (2007). Calcium signaling. *Cell* 131 1047–1058. 10.1016/j.cell.2007.11.028 18083096

[B8] Clark A. K. Old E. A. Malcangio M. (2013). Neuropathic pain and cytokines: current perspectives. *J. Pain Res.* 6 803–814. 10.2147/JPR.S53660 24294006 PMC3839806

[B9] Desai B. N. Leitinger N. (2014). Purinergic and calcium signaling in macrophage function and plasticity. *Front. Immunol.* 5:580. 10.3389/fimmu.2014.00580 25505897 PMC4245916

[B10] Epelman S. Lavine K. J. Randolph G. J. (2014). Origin and functions of tissue macrophages. *Immunity* 41 21–35. 10.1016/j.immuni.2014.06.013 25035951 PMC4470379

[B11] Fairbanks C. A. Peterson C. D. Speltz R. H. Riedl M. S. Kitto K. F. Dykstra J. A. (2014). The VGF- derived peptide TLQP-21 contributes to inflammatory and nerve injury-induced hypersensitivity. *Pain* 155 1229–1237. 10.1016/j.pain.2014.03.012 24657450 PMC4070220

[B12] Félix R. Crottès D. Delalande A. Fauconnier J. Lebranchu Y. Le Guennec J. Y. (2013). The Orai-1 and STIM-1 complex controls human dendritic cell maturation. *PLoS One* 8:e61595. 10.1371/journal.pone.0061595 23700407 PMC3659124

[B13] Hannedouche S. Beck V. Leighton-Davies J. Beibel M. Roma G. Oakeley E. J. (2013). Identification of the C3a receptor (C3AR1) as the target of the VGF-derived peptide TLQP-21 in rodent cells. *J. Biol. Chem.* 288 27434–27443. 10.1074/jbc.M113.497214 23940034 PMC3779738

[B14] Hashimoto D. Chow A. Noizat C. Teo P. Beasley M. B. Leboeuf M. (2013). Tissue-resident macrophages self-maintain locally throughout adult life with minimal contributionfrom circulating monocytes. *Immunity* 38 792–804. 10.1016/j.immuni.2013.04.004 23601688 PMC3853406

[B15] Hewavitharana T. Deng X. Soboloff J. Gill D. L. (2007). Role of STIM and Orai proteins in the store-operated calcium signaling pathway. *Cell Calcium* 42 173–182. 10.1016/j.ceca.2007.03.009 17602740

[B16] Klos A. Wende E. Wareham K. J. Monk P. N. (2013). International union of basic and clinical pharmacology. [corrected]. LXXXVII. Complement peptide C5a, C4a and C3a receptors. *Pharmacol. Rev.* 65 500–543. 10.1111/bph.12665 23383423

[B17] Levi A. Eldridge J. D. Paterson B. M. (1985). Molecular cloning of a gene sequence regulated by nerve growth factor. *Science* 229 393–395. 10.1126/science.3839317 3839317

[B18] Lin H. Zheng C. Li J. Yang C. Hu L. (2014). Ca2+ -activated K+ channel-3.1 blocker TRAM-34 alleviates murine allergic rhinitis. *Int. Immunopharmacol.* 23 642–648. 10.1016/j.intimp.2014.10.017 25466273

[B19] Lind A. L. Emami Khoonsari P. Sjödin M. Katila L. Wetterhall M. Gordh T. (2016). Spinal cord stimulation alters protein levels in the cerebrospinal fluid of neuropathic painpatients: a proteomic mass spectrometric analysis. *Neuromodulation* 19 549–562. 10.1111/ner.12473 27513633

[B20] Liu X. Wang N. Zhu Y. Yang Y. Chen X. Fan S. (2016). Inhibition of extracellular calcium influx results in enhanced IL-12 production in LPS-treated murine macrophages by downregulation of the CaMKKβ-AMPK-SIRT1 signaling pathway. *Mediat. Inflamm.* 2016:6152713. 10.1155/2016/6152713 27313401 PMC4904125

[B21] Maratou K. Wallace V. C. Hasnie F. S. Okuse K. Hosseini R. Jina N. (2009). Comparison of dorsal root ganglion gene expression in rat models of traumatic and HIV-associated neuropathic pain. *Eur. J. Pain* 13 387–398. 10.1016/j.ejpain.2008.05.011 18606552 PMC2706986

[B22] Molteni L. Rizzi L. Bresciani E. Possenti R. Petrocchi Passeri P. Ghè C. (2017). Pharmacological and biochemical characterization of TLQP-21 activation of a binding site on CHO cells. *Front. Pharmacol.* 8:167. 10.3389/fphar.2017.00167 28424618 PMC5371653

[B23] Moss A. Ingram R. Koch S. Theodorou A. Low L. Baccei M. (2008). Origins, actions and dynamic expression patterns of the neuropeptide VGF in rat peripheral and central sensory neurones following peripheral nerve injury. *Mol. Pain* 4:62. 10.1186/1744-8069-4-62 19077191 PMC2614976

[B24] Nesargikar P. N. Spiller B. Chavez R. (2012). The complement system: history, pathways, cascade and inhibitors. *Eur. J. Microbiol. Immunol.* 2 103–111. 10.1556/EuJMI.2.2012.2.2 24672678 PMC3956958

[B25] Pannell M. Labuz D. Celik M. Ö. Keye Batra J. Siegmund A. B. (2016). Adoptive transfer of M2 macrophages reduces neuropathic pain via opioid peptides. *J. Neuroinflammation* 13:262. 10.1186/s12974-016-0735-z 27717401 PMC5055715

[B26] Riedl M. S. Braun P. D. Kitto K. F. Roiko S. A. Anderson L. B. Honda C. N. (2009). Proteomic analysis uncovers novel actions of the neurosecretory protein VGF in nociceptive processing. *J. Neurosci.* 29 13377–13388. 10.1523/JNEUROSCI.1127-09.2009 19846725 PMC2801058

[B27] Ristoiu V. (2013). Contribution of macrophages to peripheral neuropathic pain pathogenesis. *Life Sci.* 93 870–881. 10.1016/j.lfs.2013.10.005 24140886

[B28] Rivolta I. Binda A. Molteni L. Rizzi L. Bresciani E. Possenti R. (2017). JMV5656, a novel derivative of TLQP-21, triggers the activation of a calcium-dependent potassium outward current in microglial cells. *Front. Cell Neurosci.* 11:41. 10.3389/fncel.2017.00041 28280458 PMC5322282

[B29] Rossol M. Pierer M. Raulien N. Quandt D. Meusch U. Rothe K. (2012). Extracellular Ca2+ is a danger signal activating the NLRP3 inflammasome through G protein-coupled calcium sensing receptors. *Nat. Commun.* 2012:1329. 10.1038/ncomms2339 23271661 PMC3535422

[B30] Sibilia V. Pagani F. Bulgarelli I. Mrak E. Broccardo M. Improta G. (2010). TLQP-21, a VGF-derived peptide, prevents ethanol-induced gastric lesions: insights into its mode of action. *Neuroendocrinology* 92 189–197. 10.1159/000319791 20805684

[B31] Skaper S. D. Giusti P. Facci L. (2012). Microglia and mast cells: two tracks on the road to neuroinflammation. *FASEB J.* 26 3103–3117. 10.1096/fj.11-197194 22516295

[B32] Soboloff J. Rothberg B. S. Madesh M. Gill D. L. (2012). STIM proteins: dynamic calcium signal transducers. *Nat. Rev. Mol. Cell Biol.* 13 549–565. 10.1038/nrm3414 22914293 PMC3458427

[B33] Vaeth M. Zee I. Concepcion A. R. Maus M. Shaw P. Portal-Celhay C. (2015). Ca2+ signaling but not store-operated Ca2+ entry is required for the function of macrophages and dendritic cells. *J. Immunol.* 195 1202–1217. 10.4049/jimmunol.1403013 26109647 PMC4506881

[B34] van den Pol A. N. Decavel C. Levi A. Paterson B. (1989). Hypothalamic expression of a novel gene product, VGF: immunocytochemical analysis. *J. Neurosci.* 9 4122–4137. 10.1523/JNEUROSCI.09-12-04122.1989 2556505 PMC6569627

[B35] Willemen H. L. Eijkelkamp N. Garza Carbajal A. Wang H. Mack M. Zijlstra J. (2014). Monocytes/Macrophages control resolution of transient inflammatory pain. *J. Pain* 15 496–506. 10.1016/j.jpain.2014.01.491 24793056 PMC4075647

[B36] Wynn T. A. Chawla A. Pollard J. W. (2013). Macrophage biology in development, homeostasis and disease. *Nature* 496 445–455. 10.1038/nature12034 23619691 PMC3725458

[B37] Yule D. I. Williams J. A. (1992). U73122 inhibits Ca2+ oscillations in response to cholecystokinin and carbachol but not to JMV-180 in rat pancreatic acinar cells. *J. Biol. Chem.* 267 13830–13835. 1629184

