# Supplementary figures and images for: STIM Proteins and Orai Ca2+ Channels Are Involved in the Intracellular Pathways Activated by TLQP-21 in RAW264.7 Macrophages
Source: Front Pharmacol. 2018 Nov 27;9:1386. doi: 10.3389/fphar.2018.01386 (PMC6277904; doi:10.3389/fphar.2018.01386)

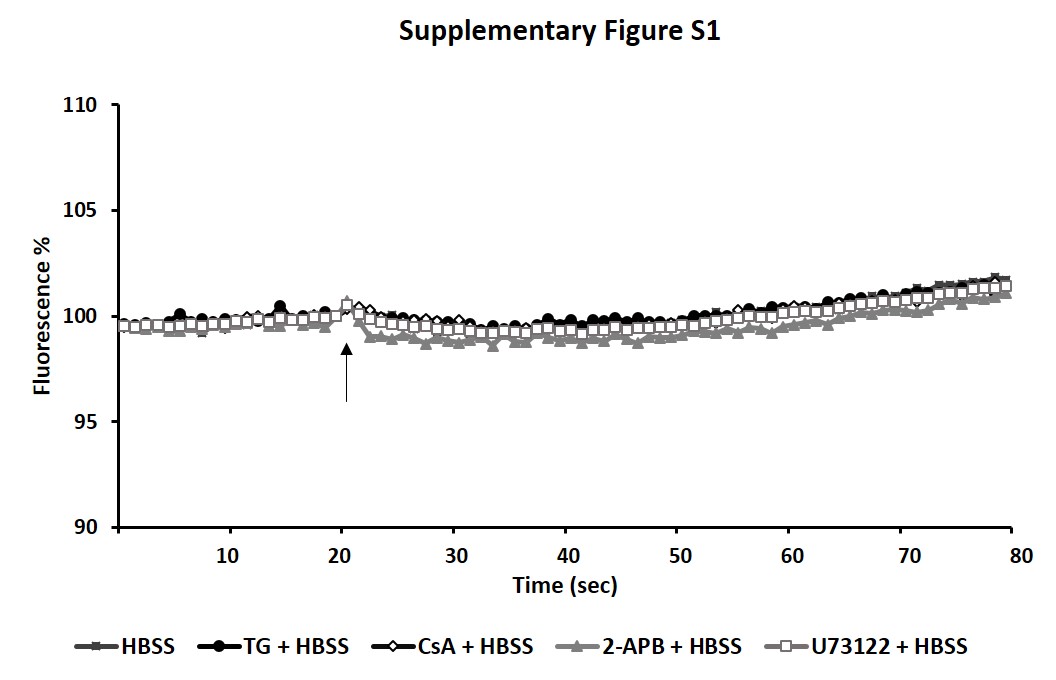

Supplement: FIGURE S1 — Effects of different inhibitors on basal fluorescence in RAW264.7 cells. Cells were loaded with FLUO-4 NW and treated with different inhibitors before the stimulation with HBSS. The lines show intracellular Ca2+ mobilization in RAW264.7 cells stimulated with HBSS alone and in presence of 2 μM CsA (15 min), 2 μM TG (20 min), 10 μM U73122 (10 min), or 75 μM 2-APB (15 min). HBSS was injected at the time indicated by the arrow. Results are the means of measurements obtained in at least six different wells for each experiment. All experiments were repeated three times. One representative experiment is shown. [file Image_1.jpg]

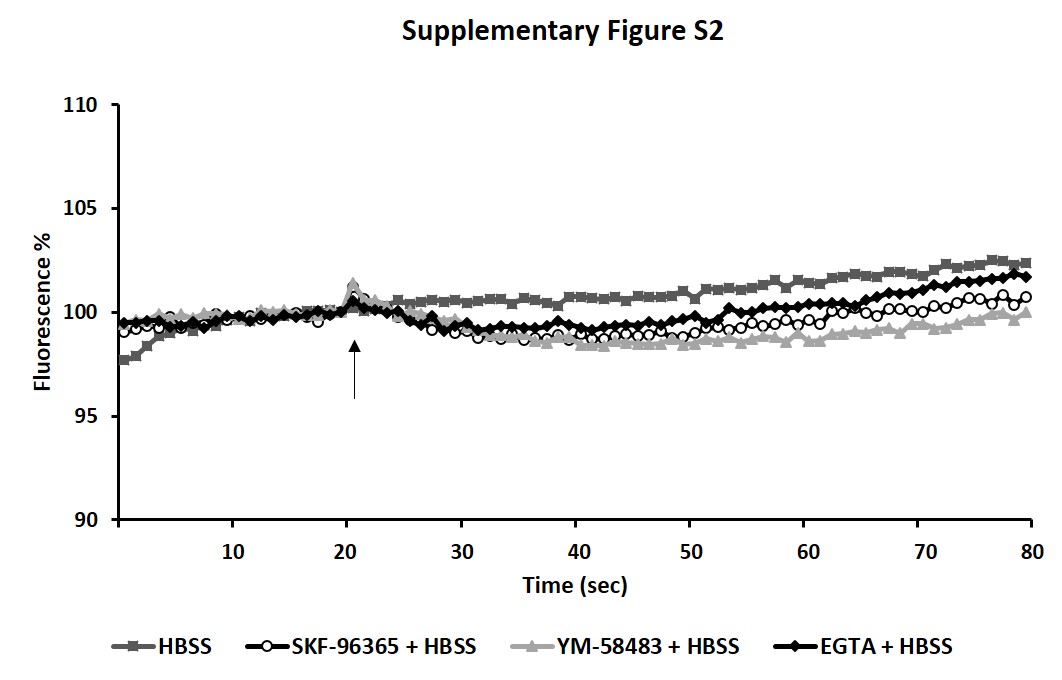

Supplement: FIGURE S2 — Modulation of basal fluorescence in the presence of SOCE antagonists in RAW264.7 cells. Cells were loaded with FLUO-4 NW and treated with different inhibitors before the stimulation with HBSS. The lines show intracellular Ca2+ mobilization in RAW264.7 cells stimulated with HBSS alone and in presence of 10 μM SKF-96365 (20 min), 10 μM YM-58483 (20 min), or 1 mM EGTA (30 min). HBSS was injected at the time indicated by the arrow. Results are the means of measurements obtained in at least six different wells for each experiments. All experiments were repeated three times. One representative experiment is shown. [file Image_2.jpg]

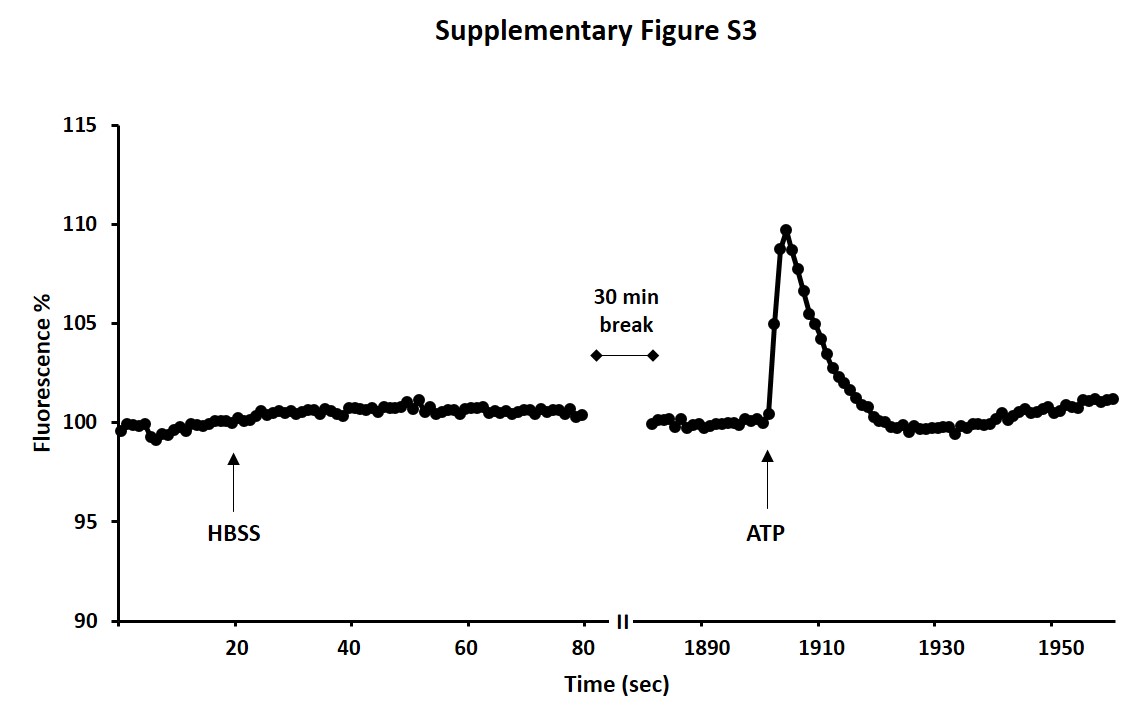

Supplement: FIGURE S3 — Effects of repeated stimulations on intracellular Ca2+ levels in RAW264.7 cells. Cells were loaded with FLUO-4 NW and fluorescence emissions were measured at 485/535 nm every 0.5 s for the 20 s preceding and the 60 s following the injection of the stimuli. 10 μM ATP was applied after 30 min from HBSS. HBSS and ATP were injected at the time indicated by the arrow. Results are the means of measurements obtained in at least six different wells for each experiments. All experiments were repeated three times. One representative experiment is shown. [file Image_3.jpg]

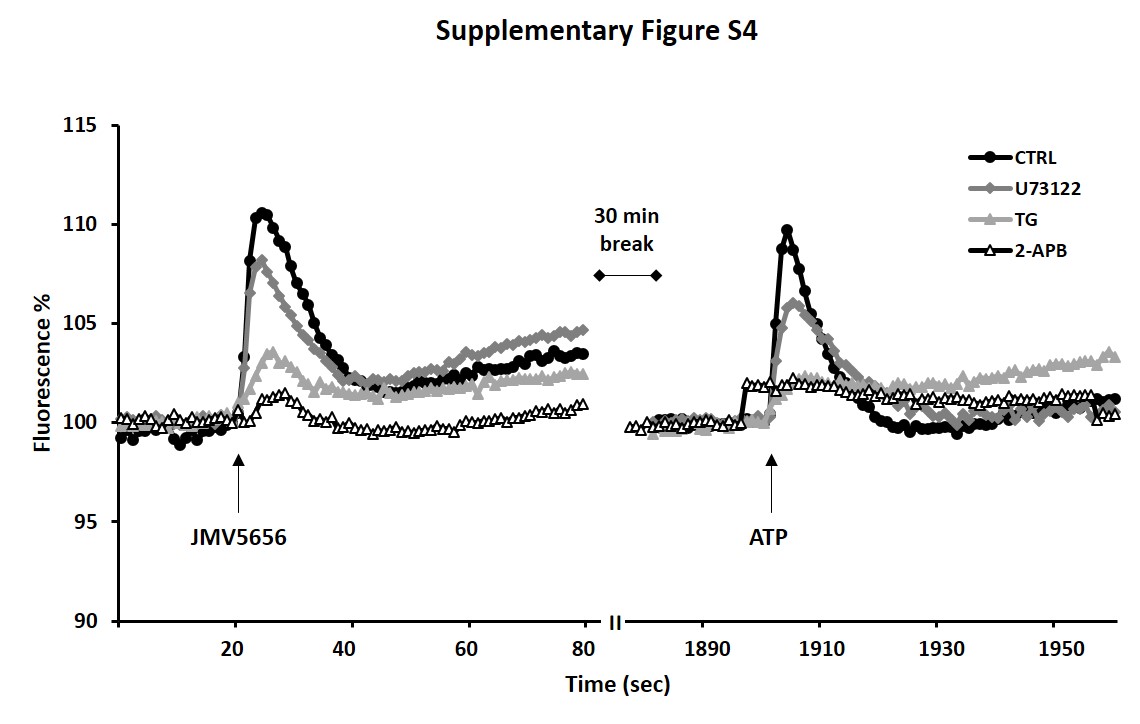

Supplement: FIGURE S4 — Repeated JMV5656 and ATP stimulations on intracellular Ca2+ levels in RAW264.7 cells. Cells were loaded with FLUO-4 NW and treated with different inhibitors (2 μM TG 20 min, 10 μM U73122 10 min, 75 μM 2-APB 15 min) before the stimulation with 1 μM JMV5656. 10 μM ATP was applied after 30 min from JMV5656. JMV5656 and ATP were injected at the time indicated by the arrow. Results are the means of measurements obtained in at least six different wells for each experiment. All experiments were repeated three times. One representative experiment is shown. [file Image_4.jpg]

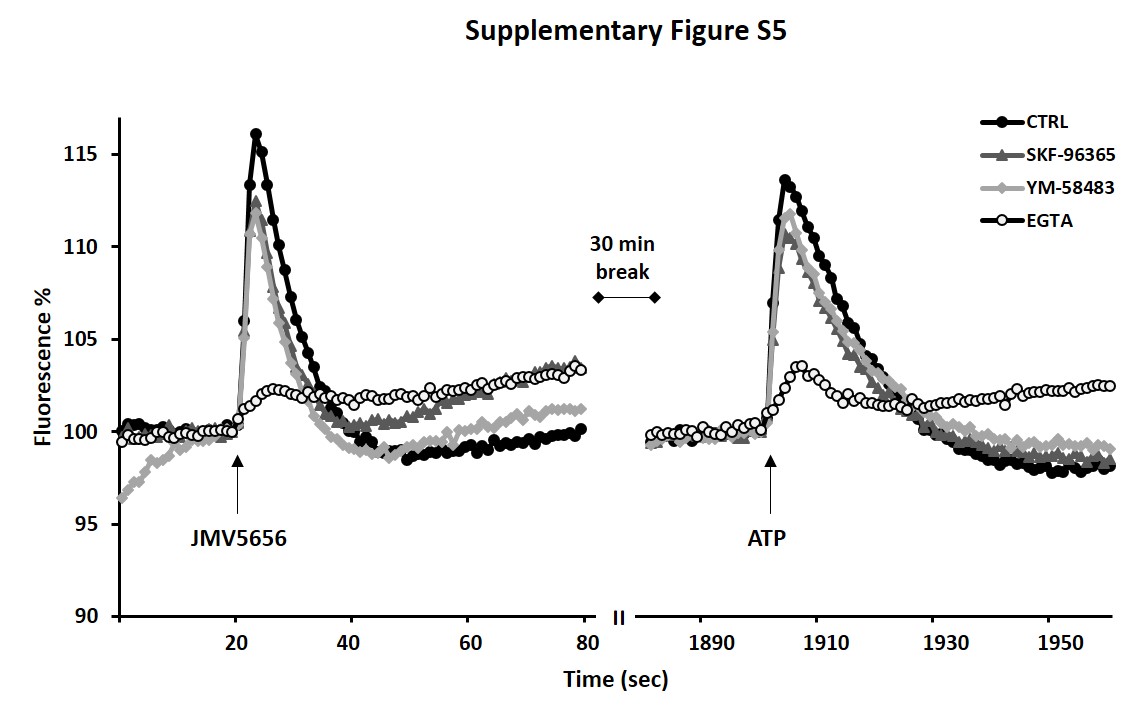

Supplement: FIGURE S5 — Repeated JMV5656 and ATP stimulations on intracellular Ca2+ levels in the presence of SOCE antagonists in RAW264.7 cells. Cells were loaded with FLUO-4 NW and treated with different inhibitors (10 μM SKF-96365 20 min, 10 μM YM-58483 20 min, 1 mM EGTA 30 min) before the stimulation with 1 μM JMV5656. 10 μM ATP was applied after 30 min from JMV5656. JMV5656 and ATP were injected at the time indicated by the arrow. Results are the means of measurements obtained in at least six different wells for each experiment. All experiments were repeated three times. One representative experiment is shown. [file Image_5.jpg]
